# Executive Function Deficit and Medial Temporal Lobe Atrophy in Late-Life Depression: Pathophysiological and Therapeutic Implications

**DOI:** 10.31083/AP51117

**Published:** 2026-07-23

**Authors:** Qianqian Zhang, Chaomeng Liu, Qinge Zhang

**Affiliations:** ^1^Beijing Key Laboratory of Mental Disorders, National Clinical Research Center for Mental Disorders & National Center for Mental Disorders, Beijing Anding Hospital, Capital Medical University, 100088 Beijing, China; ^2^Advanced Innovation Center for Human Brain Protection, Capital Medical University, 100069‌ Beijing, China

Late-life depression (LLD; depression in adults aged 65 and over) can worsen physical comorbidities, delay recovery following medical treatment, and increase suicide risk [1,2]. Unlike depression in younger populations, LLD is frequently associated with impairments across multiple cognitive domains, particularly executive function deficit (EFD), which includes difficulties with inhibitory control, working memory, and cognitive flexibility [3]. Moreover, LLD may represent a prodromal manifestation of Alzheimer’s disease (AD) [4].

AD is a progressive irreversible neurodegenerative disorder characterized by cognitive decline and behavioral impairments. Given the absence of reliable preventive or disease-modifying therapy for AD, growing attention has been drawn to its prevention through the identification of modifiable risk factors (e.g., depression [5]) in individuals at elevated risk [6]. A cohort study with a mean follow-up of 5.45 years demonstrated that adults experiencing LLD with EFD during acute episodes had an elevated risk of subsequent dementia [7]. Medial temporal lobe atrophy (MTA), especially in specific subregions such as the perirhinal cortex (Brodmann area 36), is a crucial neurobiological connection linking EFD in LLD to an increased risk of AD and should therefore be prioritized as a primary target in future diagnostic and therapeutic strategies.

According to the prevailing opinion, cognitive impairment results from interactions among multiple brain regions [8]. However, current methodologies for identifying brain network patterns linked to cognitive impairment predominantly depend on group-level analyses with multiple-comparison corrections, potentially obscuring the complex and interconnected relationships among brain regions [9]. A cross-sectional study employed a neural network–based reverse correlation method to discern connectivity features associated with cognitive impairment in late-onset depression (a major depressive episode occurring for the first time in an older person) [9]. The largest differences between late-onset depression patients with and without mild cognitive impairment were localized in the inferior, middle, and anterior regions of the left temporal pole. As the power and accessibility of machine learning models continue to increase, this method provides a practical and interpretable alternative for exploratory neuroimaging research. In addition, the abovementioned study suggested that alterations in the internal structure of the temporal lobe in individuals with late-onset depression are potential anatomical biomarkers for the early prediction of AD. Although numerous studies have investigated the hippocampus in the context of LLD, few have focused on hippocampal subfields and their interconnected medial temporal lobe regions, primarily because the hippocampus is often treated as a singular brain region despite comprising multiple structurally and functionally interconnected subregions with distinct functions and connections [10]. These connections include those to other medial temporal lobe regions, such as the entorhinal and periolfactory cortices [10]. Notably, the perirhinal cortex is the first region affected by neurofibrillary tangle pathology in the early stages of AD [11].

Taylor et al. [10] examined 59 adults with LLD and 21 age-matched controls without a history of depression (mean age = 66.4 years), revealing no notable volumetric differences in the total hippocampus or its subfields between groups. However, the LLD group demonstrated markedly reduced perirhinal cortex volumes, particularly in Brodmann area 36, a region known to undergo atrophy in prodromal AD. Previous research has documented hippocampal volume atrophy in patients with LLD [12]. Variations in findings across studies may be attributed to differences in spatial resolution, image quality, or research methodologies. Another comparative study [13] demonstrated that among adults with LLD, longer completion times on the Trail Making Test Part A were positively correlated with the severity of MTA, and patients with AD exhibited more pronounced EFD and MTA than those with LLD. These findings suggest that MTA may represent a structural marker associated with EFD in adults with LLD and thus have value for predicting future cognitive decline and dementia risk.

LLD treatment strategies predominantly rely on selective serotonin reuptake inhibitors, which are typically considered the first-line pharmacological intervention [14]. Vortioxetine may be useful for managing LLD [15], although further investigation is required to effectively address EFD in patients with LLD [16]. Psychotherapy has gained attention as an adjunct to pharmacotherapy, with cognitive behavioral therapy having the potential to mitigate EFD [17]. Nonetheless, psychotherapy outcomes are highly dependent on therapist expertise and treatment fidelity, which may limit scalability. Intermittent theta burst stimulation (iTBS), a patterned form of repetitive transcranial magnetic stimulation (rTMS), mimics endogenous theta rhythms and facilitates long-term synaptic potentiation [18]. The U.S. Food and Drug Administration (2018) approved iTBS for the treatment of adult depression [19]. A small open-label study [20] reported that iTBS applied to the left dorsolateral prefrontal cortex (DLPFC) notably alleviated depressive symptoms and improved executive function in adults with LLD, although the underlying mechanisms remain unclear.

Impaired synaptic plasticity, particularly within frontal networks, may contribute to the increased dementia risk observed in patients with LLD [21]. The prefrontal cortex plays a central role in inhibitory control. The results of neuromodulation research suggest that interventions such as electroconvulsive therapy, rTMS, and focused ultrasound share a therapeutic mechanism involving the induction of cortical plasticity [21] and may therefore mitigate frontal network plasticity deficits and exert procognitive effects when applied to LLD. Given the extensive connectivity between cortical and subcortical regions, the modulation of prefrontal circuits may also exert downstream effects on posterior regions via frontoparietal and frontotemporal pathways, potentially offering a therapeutic approach for EFD in LLD.

Despite promising findings, neuromodulation outcomes remain highly variable, which may partly be due to structural brain differences in individuals with LLD [22]. In transcranial magnetic stimulation, stimulation intensity is generally calibrated according to the motor threshold, which is ascertained by inducing motor-evoked potentials from the motor cortex [23]. The distance from the scalp to the cortex typically increases with age and differs between the motor cortex and DLPFC in conditions such as aging, dementia, and depression [24,25]. Correcting for the scalp-to-cortex distance is crucial to ensure consistent and adequate stimulation, as it accounts for interindividual variability and the frequently considerable anatomical differences between the motor cortex and DLPFC within the same individual [21].

Furthermore, heterogeneity in the organization and connectivity of large-scale brain networks may influence individual responses to neuromodulatory intervention [26,27,28].

Functional magnetic resonance imaging–based network mapping facilitates the identification of connectivity patterns, enabling the customization of stimulation targets according to an individual’s specific neuroanatomy and network organization [29]. In AD, the modulation of the parietal-lobe-to-hippocampus network through personalized rTMS has been associated with enhanced cognitive outcomes [29]. Considering the important role of the medial temporal lobe in the EFD of patients with LLD, future research involving iTBS may yield promising outcomes by focusing on the functional connectivity between the DLPFC and medial temporal lobe as a therapeutic target. These observations underscore the necessity for personalized neuromodulation strategies that customize stimulation parameters according to individual neuroanatomical and network characteristics, thereby optimizing plasticity targeted at the prefrontal cortex.
